# Analyst Site Visits and Corporate Environmental Information Disclosure: Evidence from China

**DOI:** 10.3390/ijerph192316223

**Published:** 2022-12-04

**Authors:** Linyan Fan, Sheng Yao

**Affiliations:** 1 School of Economics and Management, China University of Mining and Technology, Xuzhou 221116, China; 2 School of Management, Shanghai University, Shanghai 200444, China

**Keywords:** analyst site visits, environmental information disclosure, information asymmetry, information effect

## Abstract

Compared with developed countries, emerging economy countries are facing more severe environmental challenges. Therefore, effective disclosure of corporate environmental information is an important concern for emerging economies to cope with environmental issues. There is a growing volume of literature documenting that analyst site visits can urge corporations to provide high-quality financial information to investors. However, whether analyst site visits can also improve the quality of environmental information is still unclear. In the Chinese setting, where environmental information has attracted much attention, we explore the interaction between analyst site visits and environmental information disclosure. With three regression methods of the ordinary least squares model, two-stage least square model, and difference-in-difference model, we establish regressions to verify the relationships between them by using empirical data from 2012 to 2019 in China. The results show that analyst site visits are significantly positively correlated with corporate environmental information disclosure. This positive relation is more pronounced when corporations are in economically developed and highly market-oriented areas, in poor air quality areas, and for corporations with good, reasonable internal governance. In addition, we find that analyst site visits affect the quality of environmental information disclosure through the intermediary effect of media attention. In the robustness test, further evidence also indicates that the interaction between analyst site visits and corporate environmental information disclosure was more significant before the COVID-19 lockdown policy was implemented in Wuhan. Our findings suggest that governments should provide support for analysts to conduct site visits and formulate regulations on mandatory disclosure of environmental information by different regions as soon as possible.

## 1. Introduction

In the past, to achieve industrialization, create jobs, and reduce poverty, China, like many developing nations, paid very little attention to environmental issues, as environmental problems were usually not imminent or conspicuous when compared to the short-term goal of rapid economic development. In recent years, concerns over environmental protection have increasingly become a high-profile issue around the world [1,2,3,4]. The corporate environmental information disclosure (CEID) has become increasingly popular as an alternative environmental management approach for environmental regulation [5,6]. Its significance is to improve the transparency of corporate information, which can strengthen the communication between the public and the government and corporations so that all parties can form a consensus in the process of informed and participatory interaction. Finally, we can improve the long-term competitiveness of corporations, maintain the effective operation of the capital market, and improve environmental regulation. While the Chinese government is exploring solutions to incentivize corporations to implement CEID for improved environmental management, this enforcement of environmental regulation is still difficult to achieve due to the low level and quality of CEID in China, which has attracted public criticism [7,8,9]. In prior studies, an abundance of evidence has revealed that external public pressure from government regulation and social supervision, such as the implementation of measures for the disclosure of environmental information and government financing requirements, is an important force in driving CEID [10,11,12,13]. Current domestic regulators do not yet have standardized disclosure requirements for corporate environmental information [7,14], which suggests that inadequate access to environmental information is the main reason for China’s weak environmental management [15]. Compared to the government’s ability to rely on public power to discover and identify corporate environmental information, environmental information released by analysts as non-governmental third parties to conduct site visits has a greater marginal utility in constraining corporate behavior [16,17]. There is limited research exploring external governance mechanisms, such as the monitoring role of financial analysts, in enhancing environmental information disclosure, so our study aims to shed further light on this problem.

We selected listed corporations in polluting industries from 2012 to 2019 in China as a sample and used the ordinary least square (OLS) model, two-stage least square (2-SLS) model, and difference-in-difference (DID) model to evaluate the effect of analyst site visits on CEID. The results show that analyst site visits can significantly improve the quality of CEID. This positive relation is more pronounced when corporations are in economically developed and highly market-oriented areas, in poor air quality areas, and for corporations with good, reasonable internal governance. After considering the problem of endogeneity, the results remain robust.

Our study contributes to the literature as follows. First, study on the economic consequences of analyst site visits is extended to the non-financial domain. Earlier studies have focused on the impact of analyst site visits on information acquisition [17,18,19] and on improving the accuracy of earnings accuracy [17,20]. Within the current institutional context of China’s green development policy, our paper is distinct from existing studies as it explores the economic implications of analyst site visits across a vital aspect of environmental governance, namely CEID. Second, our paper proposes a new approach for improving CEID from the standpoint of information asymmetry. Prior research has concentrated chiefly on environmental disclosures in developed countries where the general public and other stakeholders are more aware of the significance of environmental protection during a protracted period of economic expansion. The literature seems to be quite limited with respect to public environmental disclosures in emerging markets. Previous studies on the influencing factors of CEID have focused on government administrative instruments, such as environmental regulation and government financing [11,21], and non-administrative instruments, including public opinion monitoring and public pressure [10]. Our paper provides a new and more direct means to promote corporate environmental governance from the perspective of information asymmetry with the help of the special context of analyst site visits.

The remainder of the paper is organized as follows. Section 2 reviews the literature on analyst site visits and environmental information disclosure. Section 3 forwards hypothesis. The research design and sample selection are described in Section 4, and the empirical results and robustness tests presented in Section 5. Section 6 concludes and makes suggestions

## 2. Literature Review

### 2.1. Research on Analyst Site Visits

In this section, we review the literature from the perspectives of both the researcher (analysts) and the respondent (corporations). For researchers, maintaining strong relationships with management is fundamental to their success [22], and site visit activities are becoming increasingly popular in the market [23]. Corporate field research refers to site visits by analysts to a corporate headquarters and its operational facilities. During the site visit, investors can talk with managers and other employees [17]. A two-way communication channel is provided for the site visitors and the manager. In addition to site visits, one can also have an informational advantage after communicating privately with managers via conference calls, private calls, non-deal roadshows, and face-to-face meetings [24,25,26,27,28]. Han et al. [18] examined the improvement in forecast accuracy resulting from analyst site visits to public corporations and found that the accuracy of analyst earnings forecasts for a corporation improved about 30%, on average, after visiting that corporation. Cheng et al. [17] examined the relationship between institutional site visits at public corporations and analyst earnings forecasts and found that analysts who participate in site visits have access to more private information than other analysts, which significantly improves forecast accuracy.

The recent literature has focused on the impact of analyst information acquisition activities on corporations. Chung and Jo [29] found that analyst site visits were positively related to corporate market value as well as various other proxies. In addition, these visits not only improved the information environment of the visited corporations, but also influenced the financing behavior of the visited corporations [30]. Universally, analyst site visits have a promoting impact on research in other areas of corporate governance, such as fraud detection [20], promoting corporate innovation [31], and increasing insider trading [32]. In summary, previous studies have concentrated more on the impact of analyst site visits on financial information, and few have considered the transmission of non-financial information, especially for environmental information. However, the relationship between analyst site visits and environmental information disclosure is not clear. With the growing demand for environmental information, research on whether analyst site visits, as a monitoring mechanism, increase environmental information disclosure have been quite limited.

### 2.2. Research on Corporate Environmental Information Disclosure

The determinants of CEID as a positive environmental activity of a corporation can be categorized into external and internal factors. As far as external factors are concerned, government regulation, public pressure, and peer imitation all influence the quality of CEID. In terms of government regulation, studies have shown the role of strict environmental regulation in broadly raising the importance of CEID to demonstrate the effectiveness of environmental regulation [21,33] in addition to environmental disclosure as a response of self-interested corporations to public policy pressures [34,35]. The author of [14] also reported that the CEID strategies of Chinese listed companies are oriented toward the environmental concerns of the government. In terms of public pressure, when media reports on environmental contamination increase and companies face more political and social pressures that threaten their environmental legitimacy, they try to augment environmental disclosure to demonstrate their commitment to active environmental and social responsibility and hope to win the hearts and minds of the public [36,37,38,39,40]. It has been shown that industry peers can serve as another external coercive pressure [8] and that peer imitation has a positive effect on firms’ soft environmental disclosure [41].

Considering the internal corporate factors, managers have the most truthful and holistic corporate information, and mitigating information asymmetry between stakeholders and managers is the main incentive for managers to reveal environmental information [42]. Hackston and Milne [43] investigated listed corporations in New Zealand and found that corporate size has a notable effect on environmental information disclosure. In view of the internal monitoring mechanisms of listed corporations, internal monitoring by the first largest shareholder can inhibit managers’ manipulation of environmental information disclosure [44]. Namely, the more diversified the corporate shareholding, the higher the quality of the CEID [45]. Others have attempted to perceive the impact on CEID in the light of such corporate-specific characteristics as institutional structure, the duality of the chairman and CEO (chief executive officer), high-quality audit, and analyst follow-up [35,46].

Environmental information disclosure has already become a hotpot of domestic and international research in recent years, and with environmental issues receiving constant attention and discussion, there have been some achievements of related research in this area. From the content of existing research, the factors that affect CEID are complex and indeterminate. As an important link between CEID and market, the mechanism of analyst site visits on CEID has always been a black box in related fields, and the existing research is not sufficiently deep. Therefore, our study takes the perspective of information asymmetry, which can provide a reference for effectively explaining the internal relationship between the motivating and influencing factors of CEID.

## 3. Hypotheses Development

Analyst site visits can facilitate CEID for several reasons. First, in China, the world’s largest emerging market and the second largest economic entity, environmental protection regulations are still under construction, and environmental laws and regulations are poorly implemented [39]. This yields greater discretion for corporations to disclose their environmental behavior, which increases the information asymmetry and thereby diminishes external oversight of corporate environmental governance, resulting in a lack of motivation for corporations to disclose environmental information. In addition, the ethical norms of Chinese listed corporations are still in the process of formation, and there are limited incentives for environmentally friendly behavior [47]. Therefore, besides lax government regulation, the “invisible hand” of the market can play an important and surrogate role in motivating corporations to comply with their proactive disclosure of environmental information, as consumers and other stakeholders are concerned about corporations’ environmental performance [48,49].

Second, public disclosure mechanisms in developing countries may be a useful tool when government enforcement resources are limited [50], but Chinese regulators do not yet have standardized disclosure requirements for corporate environmental information, and the increased information asymmetry causes this proven approach to be “dysfunctional”. The advantage of information obtained from analyst site visits in understanding the operation of corporations through factory visits and on-site questioning has a greater effect on macro factors [16], which provides a unique context for reducing the current situation of environmental information disclosure asymmetry in China [51,52]. Strictly speaking, analysts serve an indirect monitoring role. On the one hand, their main function is to collect, analyze, and disseminate information, which is the main channel for their monitoring effectiveness [29], and the disclosure of research information can attract the attention both of stakeholders in addition to the press and media. A large amount of media coverage can draw the attention of regulators to discipline and improve the behavior of corporations [39,53]. On the other hand, when analyst site visits reveal the environmental information of corporations, investors in the market can take disciplinary action by “voting with their feet” against corporations with poor environmental governance [49]. This negative market reaction will also put pressure on corporations [52] so that analysts can play a monitoring role. The above analyses lead to the first testable hypothesis, as follows.

**Hypothesis** **1** **(H1).** *Ceteris paribus, analyst site visits are positively correlated with the quality of CEID*.

From the above analysis, analyst site visits can alleviate environmental information asymmetry and urge corporations to actively disclose environmental information. However, the environmental information disclosure status and operational characteristics of different corporations differ [54,55], and the degree of mitigation of environmental information asymmetry by analyst site visits likewise vary [56], which ultimately affects the governance effectiveness of analyst site visits. In terms of macro factors, studies have confirmed that changes in the macroenvironment are both a source of political costs for heavily polluting corporations [57] and, at the same time, an informational advantage for analysts [16]. For example, analyst facilitation is also affected when they conduct site visits in different meteorological conditions [58] and increases in per capita income can be accompanied by reductions in traditional pollutants [59]. The author of [14] confirmed that corporations operating in the relatively economically developed eastern seaboard are more likely to disclose emissions-related data. Therefore, the level of pollution in China varies so much according to geographical location and economic development that it has a possible causal impact on analysts. We posit the following hypothesis:

**Hypothesis** **2** **(H2).** *The impact of analyst site visits on CEID are more significant in economically developed, highly market-oriented areas and in areas with poor air quality*.

In terms of corporate characteristics, analysts have a greater incentive to focus on high-quality corporations [29]. Xu et al. [56] also confirmed that analysts tend to conduct site visits to public corporations with high profitability, high-operational uncertainty, good quality disclosure, close proximity, and large size. Generally, listed corporations that have a larger number of stockholders and corporations with decentralized equity characteristics are more likely to make voluntary environmental disclosures than corporations with concentrated equity [45]. Effective board governance characteristics, such as higher board independence (with a higher degree of non-duality), are influential in the regulation of environmental performance [46]. Analyst site visits also contribute to reducing the agency costs due to the separation of ownership and operation (non-duality of chairman and CEO) [60] and constrain the corporate management behavior to make decisions that are in the long-term interests of the corporations. The above arguments lead to our following hypothesis:

**Hypothesis** **3** **(H3).** *Compared with corporations with poor internal governance quality, the effect of analyst site visits in improving CEID is more obvious in non-duality corporations with lower agency costs and lower equity concentration*.

## 4. Research Design

### 4.1. Sample Selection and Data Sources

We selected A-share corporations listed in the Shanghai and Shenzhen stock exchanges from 2012 to 2019 in polluting industries as samples. We excluded financial firms, the ST firms, and firms with missing financial data. All variables were winsorized at the 1% and 99% levels. Therefore, we were left with 9832 final observations. In Table 1, the year distribution of the sample is presented in Panel A and the industry distribution in Panel B. The financial accounting information was from the China Stock Market and Accounting Research (CSMAR) database and the WIND database. The data on provincial economic metrics were from China Statistical Yearbook. The data on environmental information disclosure and analyst research data were manually collected from corporate annual reports. The regression analysis was conducted using Stata 15.1 software (StataCorp LLC, College Station, TX, USA).

### 4.2. Measure of CEID

In China, CEID is not mandatory or explicitly required by law. Therefore, we followed the practices of previous studies [61,62,63] in defining the quality of environmental information disclosure as *EID* using a content analysis approach. Environmental information is usually disclosed in a corporate annual report, where corporations provide qualitative and quantitative information about their environmental risks. Therefore, drawing on the methodology of Zeng et al. [64], and according to the classification criteria of environmental information and Article 18 of The Measures, promulgated by the Ministry of Environmental Protection, environmental information was divided into 10 major categories: (1) investment in environmental protection; (2) government funds, finance allowance, and tax reference related to the environment; (3) disposal and treatment of waste products; (4) information about ISO certification; (5) improvement of the environment; (6) environmental policy; (7) bank loans related to environmental protection; (8) lawsuits, bounties, and penalties related to environmental protection; (9) corporate environmental protection strategies, goals, and policies; and (10) other environmental-related information. Following prior studies [61,65], we assigned different scores for the quality of disclosure. For the specific disclosure area discussed above, if a corporate annual report provided both specific environmental and monetary information, the score is 3. It is important for companies to provide useful narrative and numerical information about their environmental protection policies [66]. Numerical information is particularly important for investors to estimate expected future cash flows. If the report provided specific environmental information but no monetary information, the score is 2; if the disclosure was a generic statement of corporate environmental exposure, the score is 1; if the report contained no discussion on environmental disclosure, the score is 0. The environmental information disclosure score of each of the above 10 items was defined as *SEID*, all scores were then aggregated to obtain the total *EID* score, as shown in Equation (1). In the robustness test, we further used *EID_soft* and *EID_hard* instead of *EID* [37].

(1) $$EID=\sum_{i=1}^{n}{SEID}_{i}$$

### 4.3. Measure of Analyst Site Visits

Regarding the measure of analyst site visits, we measured this in two main ways according to existing studies [17,20]. First, we set a dummy variable for whether the corporation was being visited in the current period and defined it as *Visit_dum*. Second, we measured this as the number of times the corporation was being visited in the current year and defined it as *Visit_nmb*.

### 4.4. Measure of Other Control Variables

Considering that there are many factors affecting CEID, we drew on existing research to select the control variables for our paper at two levels [11,26]: corporation and region. Including shareholding ratio of institutional investors (*Instshr*), return on assets (*ROA*), number of years since IPO (*Age*), whether audited by a Big 4 accounting firm (*Big*4), corporate value (*TobinQ*), revenue growth rate (*Growth*), number of board meetings (*Meet_nmb*), financial leverage (*Leverage*), management shareholding proportion (*MSP*), return on equity (*ROE*), per capital gross regional product (*GDP_dum*), comprehensive utility value of regional innovation capability (*Creative*), *year*, *industry*, and *province* as control variables. The definitions for all variables are shown in Appendix A.

### 4.5. Baseline Model Design

To verify whether the environmental disclosure of listed corporations in polluting industries increased or decreased after they received at least one site visit in the current year, we established the ordinary least square regression of Equation (2) as follows.

(2) $$\begin{array}{ll} EID & =\partial_{0}+\partial_{1}\times Visit\_dum(Visit\_nmb)+\partial_{2}\times Instshr+\partial_{3}\times ROA+\partial_{4}\times Age+\partial_{5}\times Big4+\\ & \partial_{6}\times TobinQ+\partial_{7}\times Growth+\partial_{8}\times Meet\_nmb+\partial_{9}\times Leverage+\partial_{10}\times MSP+\partial_{11}\times ROE\\ & +\partial_{12}\times GDP\_dum+\partial_{13}\times Creative+\sum Year+\sum Industry+\sum Province+\varepsilon \end{array}$$

## 5. Empirical Analysis

### 5.1. Descriptive Statistics

Panel A of Table 2 shows the summary statistics for all variables. The mean of *EID* was 6.7259, and the median was 6.000, indicating that the *EID* level of listed corporations in polluting industries was generally low during the sample period. The maximum of *EID* was 19 and the minimum was 0, indicating that environmental disclosure of listed corporations varied widely. The distribution of *EID_soft* and *EID_hard* was similar to that of *EID*. The average values of *EID_soft* and *EID_hard* were 1.5242 and 5.2013, respectively. This indicates that hard disclosures were more objective and informative and, therefore, provided more verifiable, credible, and precise data than soft disclosures. The mean of *Visit_dum* was 0.3618, indicating that 36.18% of the sampled corporations in polluting industries had at least one site visit. The maximum *Visit_nmb* was 3.0445 and the minimum value was 0, indicating that the number of site visits varied widely among analysts.

Panel B of Table 2 shows the results of the different tests. Looking at both the mean and median, we found that corporations with site visits had higher *EID*, and this difference was significant at the 1% level. We also observed that the increase in *EID* was greater in the group with more site visits than that with less (significant at the 1% level).

### 5.2. Baseline Regressions

Table 3 presents the results of the estimated relation between analyst site visits and CEID as shown in Equation (2). The results show that the coefficients (α_1_) for *Visit_dum* and *EID* were positive and significant at the 1% level (0.336, T = 3.060 in Column (1); 0.329, T = 2.997 in Column (2)) for the same control variables and different *year*, *industry*, and *province* fixed effects. The results suggest that the environmental disclosure of corporations in polluting industries increased after receiving at least one site visit compared to the non-site visited corporations. To further demonstrate the dynamic effects of analyst site visits on *EID*, we used the number of analyst site visits to conduct Equation (2). According to the results of OLS regression, *Visit_nmb* was positively related to *EID* at the 5% significance level, and the regression coefficients were 0.147 and 0.157, which indicates that analyst site visits can effectively improve CEID. As the *province* fixed variables were added, R^2^ also gradually increased, the explanation of Equation (2) gradually increased, and there was a significant positive correlation between them. In general, analyst site visit was positively related to CEID, verifying H1.

### 5.3. The Influence of Macroscopic and Microscopic Factors

In this section, we investigated the effects under different external and internal factors. As mentioned earlier, we hypothesized for macroscopic factors that the improvement effect of analyst site visits on CEID is facilitated in areas with higher GDP per capita [50], high marketization [67], and poor air quality [68]. For the measurement of air quality index (*AQI*), we acquired the daily *AQI* for each city in China from the official website of the Ministry of Ecology and Environment of the People’s Republic of China (MEEPC). These data are generated from daily air quality reports issued by environmental protection province- and city-level bureaus. The *AQI* is constructed based on the levels of six atmospheric pollutants: sulfur dioxide (SO_2_), nitrogen dioxide (NO_2_), suspended particulates smaller than 10 μm in aerodynamic diameter (PM_10_), suspended particulates smaller than 2.5 μm in aerodynamic diameter (PM_2.5_), carbon monoxide (CO), and ozone (O_3_). Prior to 2014, the Chinese government monitored only SO_2_, NO_2_, and PM_10_, which was used to construct the air pollution index (API) that served as a summary measure of air quality in earlier years [69]. While the API and *AQI* are not directly comparable, they are highly correlated [70]. For notational simplicity, we refer to both as *AQI* in what follows. For a small portion of the city’s daily observations, the *AQI* was not available through the MEEPC, so we used the Air Quality Standard (GB3095-2012) by the Ministry of Environmental Protection (MEP) to calculate the indexes. A previous study has shown that when analyst site visits are conducted under different meteorological conditions, their promoting effect is also affected [58], and we collected daily municipal-level *AQI* data published by MEEPC to match the date of analyst site visits. Daily meteorological data were obtained from 194 international weather stations in China provided by the China Integrated Weather Information Service (CIMIS), and we matched each city to the nearest weather station based on linear distance. Based on the location of the visited corporation and the analyst’s organizations as well as the date of the site visit, we defined *AQI* as 1 if the analyst visited a high-quality area to a low-quality area and 0 otherwise. Panel A of Table 4 reports the regression results of Equation (1) based on the external macro factor group. The results show the regression coefficients of *Visit_nmb*×*GDP_dum*, *Visit_nmb*×*Market*, and *Visit_nmb*×*AQI* were 0.216, 0.055, and 0.566, respectively, which were significant at either the 1% or 5% levels. The regression results indicate that the improvement effect of analyst site visits on CEID was more significant in economically developed and highly market-oriented areas, promoting CEID in poor air quality areas. Thus, H2 was verified.

The impact of corporate characteristics on CEID is equally important, and the differences between corporations leads to heterogeneity. Based on the above hypothesis, we selected *Duality* [46], *Shrcr10*, and *Agent* [60] as micro factors. As shown in Table 4 of Panel B, the regression coefficients of *Visit_nmb* × *Duality*, *Visit_nmb* × *Shrcr10*, and *Visit_nmb* × *Agent* were −0.719, −0.036, and −4.616, respectively, which were significant at the 1% level. This indicates that the effect of analyst site visits on CEID was more significant when the agency cost was lower, the degree of non-duality was higher, and the concentration of equity was lower. Thus, H3 was verified.

### 5.4. Mechanism Analysis

As an important external corporate governance mechanism, media attention plays a supervisory role in corporate business behavior [53,71]. Similarly, as an information intermediary [72], the media are inevitably influenced by professional analysts when mining and reporting corporate information. Numerous studies have also demonstrated that the most convenient way for analysts to obtain corporate information is still through the media [73], implying that the mining and dissemination of corporate information by analysts can increase the public information available to the media. On the one hand, previous literature also confirms that media attention plays an equally important role in corporate environmental governance [10,74], and the strong pressure of media attention on public opinion can monitor corporations toward improving the quality of their environmental disclosures and, thus, their image in regulating environmental mismanagement [75,76,77]. On the other hand, the external public pressure caused by media attention leads to government intervention. The government is bound to take active measures to regulate the environment and promulgate relevant regulations and penalties. The “political cost” generated by the regulatory authorities will affect corporations in seeking to avoid risks and disclose environmental information in detail [53,78]. We conjecture that the information disclosed by analysts after a site visit can catch the interest of stakeholders and the news media, and that induced the external public pressure can be transmitted to corporations through regulatory departments, which will constrain corporate behavior and thus influence the CEID. To test this transmission path, we used media attention (*Media*) and set Equations (3)–(5) following Wen and Ye [79].

(3) $$EID=\alpha_{0}+\alpha_{1}Visit\_nmb+\alpha_{2}Controls+\varepsilon$$

(4) $$Media=\beta_{0}+\beta_{1}Visit\_nmb+\beta_{2}Controls+\varepsilon$$

(5) $$EID=\gamma_{0}+\gamma_{1}Visit\_nmb+\gamma_{2}Media+\gamma_{3}Controls+\varepsilon$$

The measure of *Media* is consistent with the method used in prior literature [80,81,82], wherein we used the natural logarithm of the annual number of relevant media reports plus 1 to measure the level of media attention the corporation receives, which lowers the magnitude of potential noise. The annual media report data were obtained from the “Financial News Database of Chinese Listed Companies” of Chinese Research Data Services (CNRDS). The database was based on news reports from more than 400 important online media as the data pool, and relevant news content was searched by taking the corporate stock code, stock abbreviation, corporation full name, and corporation abbreviation as keywords and merging the total number of media reports for each listed corporation according to the corresponding year.

If the regression coefficients *α*_1_, *β*_1_, and *γ*_2_ were all significant and the significance level or value of *γ*_1_ decreased compared to *α*_1_, this indicates that there is a mediating effect. The results in column (2) of Table 5 show that analyst site visits have increased the number of media reports (0.379 with T = 14.214). The results of column (3) show that the regression coefficient of the effect of media attention on CEID was significantly positive, and the regression coefficient of the effect of analyst site visit on CEID was still significantly positive and lower than that of column (1). The Sobel test result was significant at the 1% level. The results show that the intermediary effect of media attention was significant, and so our expected impact path was supported.

### 5.5. Robustness Check

#### 5.5.1. Redefinition of the Dependent Variable

Aerts and Cormier [77] divided corporate environmental information disclosure into economic and social environmental information disclosure. We examined the impact of different types of environmental information on analyst site visits. The 10 *EID* score items were divided into two categories according to their types: soft information (*EID_Soft*) and hard information (*EID_Hard*). Table 6 reports the results of Equation (2) in which we replaced *EID* with *EID_soft* and *EID_hard*. The coefficients of *Visit_dum* were significantly positive at the level of 1%, and the coefficients of *Visit_nmb* were significant at the levels of 1% and 5%. The above robustness test results were consistent with the previous results, and study results remained valid.

#### 5.5.2. Endogenous Test

To further verify the robustness of our results, we considered the endogeneity problem of analyst site visits. The further away a corporation is from a regulator, the harder it is for the regulator to verify the information and the easier it is for management to manipulate the environmental information. Therefore, the quality of CEID is inevitably compromised. In order to solve the endogeneity problem, we developed a 2SLS model using the geographical distance between analyst organizations and listed corporations as an instrumental variable. As shown in Table 7, the *Distance* coefficient in the first stage was −0.245 (significantly positive at the 1% level), indicating that an analyst makes more site visits when their organization was closer to the listed corporation. In the second stage, the regression coefficient of *EID* was 6.422 (significantly positive at the 1% level), indicating that after considering the endogeneity problem, analyst site visits still had a significant positive effect on CEID. The results remained robust after accounting for endogeneity issues.

#### 5.5.3. Propensity Scores Matching Test

In order to further ensure the robustness of the empirical results, we used propensity score matching (PSM) to reselect the samples. Panel A of Table 8 shows that after one-to-one matching, the standardized deviations of all covariates were less than 10%, indicating they pass the balance test. As shown in Panel B of Table 8, the coefficients of the baseline regressions were all positively significant at the 1% level based on the matched sample. These results were consistent with the results in Table 4. It was verified that analyst site visits significantly improve the quality of CEID and ensure the robustness of the empirical results.

#### 5.5.4. The Impact of COVID-19 on the Relationship between Analyst Site Visits and CEID in Wuhan

Since early 2020, the global spread of COVID-19 has caused a major impact on countries [83]. Meanwhile, China quickly implemented an effective lockdown policy at the end of January 2020, restricting the impact of the epidemic mainly within Wuhan. Information plays a vital role in mitigating the negative impact of the novel coronavirus. However, all types of traffic in Wuhan were blocked, which makes the information channel for analysts to obtain private information through site visits limited by the traffic control and the reduction in travel of analysts during the COVID-19 outbreak. The information advantage of analysts is limited, which increases the environmental information asymmetry and affects the quality of CEID, but at the same time, it also provides us with a distinct control groups and treatment groups. At the outbreak point, the actual operational activities of each listed corporation ended in 2019, and the point of confirmation of the human-to-human infection route on 20 January 2020, also provides clearer division of the period into pre- and post-epidemic [84]. Unlike other policies, the COVID-19 as a major emergency has a high exogenous nature, while China’s more effective containment and isolation means ensured that the early impact of the epidemic was mainly contained within Wuhan [85]. Therefore, the impact of analyst site visits on the quality of CEID before and after COVID-19 can be used as reliable evidence to assess whether analysts effectively reduced environmental information asymmetry. We selected A-share companies in the heavy pollution industry affected by COVID-19 from 1 January 2018, to 31 December 2020, and 20 January 2020 was used as the treatment time point (*Post*), and listed companies registered or with offices in Wuhan city were used as the treatment group to construct the DID model to test the previous hypothesis. *Treatment* equaled 1 if a corporation belonged to a treatment group and 0 otherwise. *Post* equaled 1 if a corporation’s data came from the post-treatment period and 0 otherwise. The DID regression Equation (3) is as follows.

(6) $$EID=\alpha_{1}Treatment+\alpha_{2}Post+\alpha_{3}Treatment\times Post+controls+\varepsilon$$

We concentrated on the coefficient *α*_3_ of *Treatment×Post* in the regressions. Table 9 reports the regression results of the DID Equation (3). The results show that the coefficients (*α*_3_) on *Treatment×Post* had negative significant (−1.618, T = −8.657 in Column (1); −2.360, T = −10.797 in Column (2); −2.187, T = −10.986 in Column (3); −1.738, T = −5.909 in Column (4)) effects on the different control variables and the *year*, *industry*, and *province* fixed effects. The results suggest that after the implementation of lockdown in Wuhan, the CEID decreased in corporations in Wuhan compared with other cities in China. In other words, the CEID cannot significantly improve without analyst site visits.

## 6. Conclusions

In our paper, using analyst site visit data under the institutional background of China, we selected A-share corporations in polluting industries listed in Shanghai and Shenzhen stock exchanges from 2012 to 2019 as samples to obtain new insights regarding the relationship between analysts site visits and CEID. The results of our study show that analyst site visits significantly promoted CEID. This positive effect was more evident in economically developed regions and in corporations with better internal corporate governance. After a series of robustness tests, the above findings remained the same. Our research suggests that analyst site visits are a useful practice for environmental governance in China since they can effectively enhance the environmental awareness of local governments and firms and encourage public participation in environmental protection. Despite the fact that China is where our evidence comes from, its contributions and practical implications should not be undervalued. Environmental pollution is a global concern, from this viewpoint, the findings of this study may have significant implications not only for China but also for other countries, especially emerging economies and developing countries where environmental pollution is more serious.

This study offers important theoretical implications. From the perspective of information asymmetry, our paper enriches the research on the influencing factors of CEID. Previous scholars seldom paid attention to the impact of non-routine environmental regulation on environmental disclosure. Focusing on analyst site visits, our paper fills this gap by studying the impact of non-governmental third parties on CEID.

From the perspective of the analysts, our paper extends the field of research on the economic consequences of analyst site visits to the non-financial domain and has important practical significance for the in-depth understanding and promotion of corporate environmental governance. Most studies have focused on the impact of analyst site visits on information acquisition and on improving the accuracy of earning management. Our paper evaluated the effect of the analyst site visits through empirical tests, which not only expands the related empirical research but also provides a new perspective for further research on analyst site visits. Additionally, our research develops an analysis methodology that integrates macro-environmental factors with micro-corporation characteristics to accurately detect the overall impact of analyst site visits.

Our paper has positive policy implications. One is to make use of analyst site visits to promote CEID. We confirmed that analyst site visits have the function of promoting CEID; therefore, analysts should be actively encouraged to visit heavily polluting corporations and ask specific questions. Second, the degree of environmental information asymmetry should be fundamentally reduced. In our study, we demonstrated that the availability of environmental information helps stakeholders monitor corporate environmental governance. Considering the significant differences in regional development in China, a top-down “one-size-fits-all” approach is inadvisable. For the purpose of compelling corporations to disclose environmental information, policies and regulations should be formulated and introduced in different regions as soon as possible, enriching the level and quality of CEID by improving the standards.

There are some limitations in our study. First, the paper only used the listed corporations in the heavy pollution industry with EID data from 2012–2019 in Shenzhen and Shanghai stock exchanges as the sample, and the data was judged according to the evaluation system designed by the content evaluation method, excluding the samples with missing data. That is, the sample size does not cover the whole heavily polluting industry. A more comprehensive and accurate measurement method should be used in future studies. Second, despite the fact that this study attempted to use multiple econometric models to study the impact of analyst site visits on CEID, there are still some limitations, which are worthy of further study in the future. For example, dynamic effects were ignored in the empirical analysis, which limits the practical significance of this study to a certain extent. Nevertheless, this study has achieved its initial purpose and obtained various interesting findings.

## Figures and Tables

**Table 1:** Sample distribution across years and industry classification.

Panel A: Distribution across years									
Year	2012	2013	2014	2015	2016	2017	2018	2019	Total
Obs.	211	299	303	316	344	351	340	316	2480
Percent	8.51%	12.06%	12.22%	12.74%	13.87%	14.15%	13.71%	12.74%	100%
**Panel B** **: Distribution across industries**									
			Obs.	Percent				Obs.	Percent
Ferrous metals mining and dressing			6	0.24%	Manufacturers of clothes and other fibers products			73	2.94%
Extraction of petroleum and natural gas			8	0.32%	Building decoration and other construction industry			92	3.71%
Oil processing and refining			13	0.52%	Foodstuff manufacturing			95	3.83%
Manufacturing of leather, fur, feather, and other products			15	0.60%	Power and heat production and supply industries			107	4.31%
Mining and washing of coal industry			19	0.77%	Metal products			167	6.73%
Non-ferrous metals mining and dressing			30	1.21%	Non-ferrous metals foundries and presses			184	7.42%
Mining auxiliary activity			38	1.53%	Non-metal products			217	8.75%
Ferrous metal foundries and presses			65	2.62%	Chemical material and products manufacturing			581	23.43%
Paper making and paper products			66	2.66%	Pharmaceutical manufacturing			632	25.48%
Beverage manufacturing			72	2.90%	Total			2480	100%

**Table 2:** Descriptive statistics.

Panel A: Full sample											
Variable		Mean	Std. Dev.		Minimum	P25	Median		P75		Maximum
*EID*		6.7259	4.8750		0.0000	3.0000	6.0000		10.0000		19.0000
*EID_Soft*		1.5242	1.5442		0.0000	0.0000	1.0000		2.0000		6.0000
*EID_Hard*		5.2013	3.9898		0.0000	2.0000	5.0000		8.0000		15.0000
*Visit_dum*		0.3618	0.4806		0.0000	0.0000	1.0000		1.0000		1.0000
*Visit_nmb*		0.5671	0.8595		0.0000	0.0000	0.0000		1.0986		3.0445
*Instshr*		0.3413	0.2624		0.0000	0.0719	0.3440		0.5536		0.8930
*ROA*		0.0427	0.0610		−0.1654	0.0079	0.0345		0.0727		0.2351
*Age*		1.5748	0.7283		0.3000	1.0000	1.5000		2.2000		2.8000
*Big4*		0.0598	0.2372		0.0000	0.0000	0.0000		0.0000		1.0000
*TobinQ*		2.0208	1.4191		0.5990	1.0768	1.5604		2.4266		8.1710
*Growth*		0.1402	0.3606		−0.4926	−0.0147	0.0767		0.2195		2.3539
*Meet_nmb*		9.3380	3.6167		4.0000	7.0000	9.0000		11.0000		22.0000
*Leverage*		0.4157	0.2136		0.0465	0.2414	0.4033		0.5740		0.9518
*MSP*		0.1233	0.1950		0.0000	0.0003	0.0011		0.2234		0.6722
*Market*		7.7091	1.9137		0.6200	6.6200	7.9300		9.3500		9.7800
*GDP_dum*		0.5013	0.5000		0.0000	0.0000	1.0000		1.0000		1.0000
*Creative*		30.2818	9.2481		15.7800	25.0700	28.3500		30.8700		62.1400
**Panel B: Test for differences**											
Variable	Group			Obs.	Means	T-test of diff. in means		Medians		Wilcoxon test of diff. in medians	
*EID*	Non site visit			7352	4.184	2.113 *** (18.196)		2.000		4.000 *** (23.223)	
	Site visit			2480	6.297			6.000			
	Less site visit			7896	4.340	1.912 *** (14.993)		2.000		4.000 *** (19.566)	
	More site visit			1936	6.252			6.000			

Note: Parentheses are t-statistics based on standard errors. *** denote statistical significance at the 1% levels.

**Table 3:** Regression results of analyst site visits on CEID.

Dep. EID	(1)	(2)	(3)	(4)
*Visit_dum*	0.336 ***	0.329 ***		
	(3.060)	(2.997)		
*Visit_nmb*			0.147 **	0.157 **
			(2.419)	(2.569)
*Instshr*	2.955 ***	2.915 ***	2.968 ***	2.921 ***
	(13.471)	(13.223)	(13.505)	(13.227)
*ROA*	6.421 ***	5.709 ***	6.450 ***	5.702 ***
	(6.946)	(6.164)	(6.955)	(6.134)
*Age*	1.017 ***	1.009 ***	1.011 ***	1.005 ***
	(15.046)	(14.773)	(14.971)	(14.718)
*Big4*	−0.031	0.258	−0.040	0.251
	(−0.155)	(1.259)	(−0.199)	(1.224)
*TobinQ*	−0.301 ***	−0.289 ***	−0.298 ***	−0.286 ***
	(−8.295)	(−7.991)	(−8.211)	(−7.912)
*Growth*	−0.280 *	−0.246	−0.277 *	−0.244
	(−1.762)	(−1.557)	(−1.744)	(−1.545)
*Meet_nmb*	0.269 ***	0.274 ***	0.271 ***	0.275 ***
	(23.683)	(23.929)	(23.852)	(24.084)
*Leverage*	−0.384 *	−0.363 *	−0.306	−0.300
	(−1.838)	(−1.732)	(−1.493)	(−1.457)
*MSP*	2.627 ***	2.567 ***	2.680 ***	2.607 ***
	(9.177)	(8.960)	(9.399)	(9.138)
*Market*	−0.068 **	−0.042	−0.067 **	−0.041
	(−2.202)	(−0.874)	(−2.176)	(−0.856)
*GDP_dum*	0.061	0.014	0.057	0.013
	(0.527)	(0.084)	(0.487)	(0.082)
*Creative*	0.002 **	0.002 *	0.002 **	0.002 *
	(2.106)	(1.710)	(2.120)	(1.716)
*Constant*	3.469 ***	2.356 ***	3.464 ***	2.346 ***
	(8.468)	(4.392)	(8.444)	(4.372)
*Year fixed effect*	Yes	Yes	Yes	Yes
*Industry fixed effect*	Yes	Yes	Yes	Yes
*Province fixed effect*	No	Yes	No	Yes
*Observations*	9824	9824	9824	9824
*Adj.R2*	0.426	0.436	0.426	0.436

Note: Parentheses are t-statistics based on standard errors. ***, **, and * denote statistical significance at the 1%, 5%, and 10% levels, respectively.

**Table 4:** Regression results of macroscopic and microscopic influencing factors.

Panel A: The moderating effect of macroscopical influencing factors						
Dep. EID		GDP_dum		Market		AQI
*Visit_nmb*		0.120		0.068		−0.165
		(1.397)		(0.226)		(−1.305)
*GDP_dum*		−0.066				
		(−0.368)				
*Visit_nmb* *×GDP_dum*		0.216 **				
		(1.970)				
*Market*				−0.124		
				(0.561)		
*Visit_nmb* *×Market*				0.055 *		
				(2.192)		
*AQI_dum*						−0.119
						(−0.260)
*Visit_nmb* *×AQI*						0.566 *
						(2.028)
*Controls*		Yes		Yes		Yes
*Year fixed effect*		Yes		Yes		Yes
*Industry fixed effect*		Yes		Yes		Yes
*Province fixed effect*		Yes		Yes		Yes
*Observations*		9824		9824		9824
*Adj.R^2^*		0.362		0.384		0.487
**Panel B: The moderating effect of microcosmic influencing factors**						
Dep. EID	Duality		Shrcr10		Agent	
*Visit_nmb*	0.350 ***		2.284 ***		0.503 ***	
	(5.030)		(10.665)		(4.756)	
*Duality*	0.729 ***					
	(5.316)					
*Visit_nmb* *×Duality*	−0.719 ***					
	(−5.857)					
*Shrcr10*			0.071 ***			
			(29.354)			
*Visit_nmb* *×Shrcr10*			−0.036 ***			
			(−10.622)			
*Agent*					8.006 ***	
					(7.635)	
*Visit_nmb* *×Agent*					−4.616 ***	
					(−4.384)	
*Controls*	Yes		Yes		Yes	
*Year fixed effect*	Yes		Yes		Yes	
*Industry fixed effect*	Yes		Yes		Yes	
*Province fixed effect*	Yes		Yes		Yes	
*Observations*	9824		9824		9824	
*Adj.R^2^*	0.438		0.482		0.439	

Note: Parentheses are t-statistics based on standard errors. ***, **, and * denote statistical significance at the 1%, 5%, and 10% levels, respectively.

**Table 5:** Mechanism analysis results of the analyst site visits on CEID.

Dep.	(1)	(2)	(3)
	EID	Media	EID
*Visit_nmb*	0.157 **	0.379 ***	0.112 *
	(2.569)	(14.214)	(1.818)
*Media*			0.118 ***
			(5.111)
*Instshr*	2.921 ***	1.605 ***	2.731 ***
	(13.227)	(16.652)	(12.210)
*ROA*	5.702 ***	−0.861 **	5.804 ***
	(6.134)	(−2.123)	(6.250)
*Age*	1.005 ***	1.280 ***	0.853 ***
	(14.718)	(42.981)	(11.473)
*Big4*	0.251	−0.156 *	0.270
	(1.224)	(−1.744)	(1.316)
*TobinQ*	−0.286 ***	0.229 ***	−0.313 ***
	(−7.912)	(14.523)	(−8.581)
*Growth*	−0.244	0.077	−0.253
	(−1.545)	(1.113)	(−1.604)
*Meet_nmb*	0.275 ***	0.102 ***	0.263 ***
	(24.084)	(20.491)	(22.574)
*Leverage*	−0.300	0.101	−0.312
	(−1.457)	(1.120)	(−1.517)
*MSP*	2.607 ***	2.563 ***	2.304 ***
	(9.138)	(20.583)	(7.914)
*Market*	−0.041	0.019	−0.057
	(−0.856)	(0.725)	(−0.949)
*GDP_dum*	0.013	0.099	0.001
	(0.082)	(1.409)	(0.009)
*Creative*	0.002 *	−4.872 × 10^−4^	0.002 *
	(1.716)	(−1.255)	(1.783)
*Constant*	2.346 ***	0.241	−0.396
	(4.372)	(0.524)	(−0.376)
*Sobel test*			0.045 ***
			(4.810)
*Year fixed effect*	Yes	Yes	Yes
*Industry fixed effect*	Yes	Yes	Yes
*Province fixed effect*	Yes	Yes	Yes
*Observations*	9824	9824	9824
*Adj.R^2^*	0.436	0.624	0.437

Note: Parentheses are t-statistics based on standard errors. ***, **, and * denote statistical significance at the 1%, 5%, and 10% levels, respectively.

**Table 6:** The regression results of replacing the dependent variables.

Dep.	EID_Soft		EID_Hard	
	(1)	(2)	(1)	(2)
*Visit_dum*	0.099 ***		0.235 ***	
	(2.786)		(2.609)	
*Visit_nmb*		0.060 ***		0.100 **
		(3.015)		(1.999)
*Controls*	Yes	Yes	Yes	Yes
*Year fixed effect*	Yes	Yes	Yes	Yes
*Industry fixed effect*	Yes	Yes	Yes	Yes
*Province fixed effect*	Yes	Yes	Yes	Yes
*Observations fixed effect*	9824	9824	9824	9824
*Adj.R^2^*	0.291	0.291	0.412	0.412

Note: Parentheses are t-statistics based on standard errors. *** and ** denote statistical significance at the 1% and 5% levels, respectively.

**Table 7:** Two-stage regression results based on the instrumental variable method.

Dep.	First Stage	Second Stage
	*Visit_nmb*	*EID*
*Visit_nmb*		6.422 ***
		(4.800)
*Distance*	−0.245 ***	
	(−6.488)	
*Instshr*	0.548 ***	−0.319
	(13.96)	(−0.397)
*ROA*	2.437 ***	−8.475 **
	(14.92)	(−2.378)
*Age*	−0.0932 ***	1.594 ***
	(−7.708)	(9.416)
*Big4*	−0.186 ***	1.484 ***
	(−5.088)	(3.649)
*TobinQ*	0.0176 ***	−0.411 ***
	(2.743)	(−6.763)
*Growth*	0.0641 **	−0.696 ***
	(2.246)	(−2.630)
*Meet_nmb*	0.0202 ***	0.156 ***
	(10.03)	(4.693)
*Leverage*	0.975 ***	−6.690 ***
	(27.17)	(−4.831)
*MSP*	0.806 ***	−2.282 *
	(15.95)	(−1.948)
*Market*	0.0843 ***	−1.056 ***
	(3.382)	(−4.359)
*GDP_dum*	−0.0558 *	0.161
	(−1.752)	(0.569)
*Creative*	0.000165	0.000359
	(1.018)	(0.251)
*Constant*	−0.882 ***	18.79 ***
	(−3.629)	(7.157)
*Year fixed effect*	Yes	Yes
*Industry fixed effect*	Yes	Yes
*Province fixed effect*	Yes	Yes
*Observations*	8593	8593
*Adj.R^2^*	0.310	0.388

Note: Parentheses are t-statistics based on standard errors. ***, **, and * denote statistical significance at the 1%, 5%, and 10% levels, respectively.

**Table 8:** Propensity score matching test results.

Panel A: Propensity score matching balance test results.										
Var.	Unmatched		Mean			Bias (%)		*t*-test		
	Matched		Treated		Control			t		P > T
*Instshr*	U		0.322		0.210	42.8		18.28		0.000
	M		0.322		0.329	−2.7		−0.95		0.341
*Big4*	U		0.036		0.044	−4.0		−1.67		0.094
	M		0.036		0.032	−2.1		0.78		0.433
*Growth*	U		0.172		0.066	38.1		17.19		0.000
	M		0.172		0.170	0.7		0.20		0.842
*Leverage*	U		0.274		0.103	84.1		37.66		0.000
	M		0.274		0.279	−2.7		−0.80		0.425
*MSP*	U		0.191		0.054	75.0		36.16		0.000
	M		0.191		0.186	2.9		0.83		0.407
*Market*	U		8.014		7.915	5.4		2.27		0.024
	M		8.014		7.865	8.1		2.87		0.004
**Panel B: Regression results of analyst site visits and EID using matched sample.**										
Dep. EID		(1)		(2)			(3)		(4)	
*Visit_dum*		0.548 ***		0.460 ***						
		(3.777)		(3.158)						
*Visit_nmb*							0.284 ***		0.258 ***	
							(3.722)		(3.355)	
*Instshr*		2.074 ***		1.703 ***			2.030 ***		1.652 ***	
		(6.051)		(4.914)			(5.906)		(4.752)	
*ROA*		5.659 ***		4.978 ***			5.450 ***		4.717 ***	
		(4.035)		(3.562)			(3.859)		(3.349)	
*Age*		1.243 ***		1.141 ***			1.248 ***		1.146 ***	
		(9.990)		(8.972)			(10.025)		(9.008)	
*Big4*		−0.354		−0.060			−0.363		−0.066	
		(−1.002)		(−0.169)			(−1.027)		(−0.186)	
*TobinQ*		−0.466 ***		−0.457 ***			−0.457 ***		−0.449 ***	
		(−8.322)		(−8.135)			(−8.183)		(−8.012)	
*Growth*		−0.455 **		−0.385 *			−0.464 **		−0.392 *	
		(−2.010)		(−1.717)			(−2.048)		(−1.747)	
*Meet_nmb*		0.157 ***		0.154 ***			0.158 ***		0.155 ***	
		(8.502)		(8.307)			(8.629)		(8.411)	
*Leverage*		−0.227		−0.320			−0.166		−0.281	
		(−0.735)		(−1.029)			(−0.541)		(−0.908)	
*MSP*		1.762 ***		1.210 ***			1.768 ***		1.206 ***	
		(4.485)		(3.027)			(4.502)		(3.018)	
*Market*		−0.073		−0.171 *			−0.077		−0.174 *	
		(−1.341)		(−1.868)			(−1.399)		(−1.901)	
*GDP_dum*		−0.123		0.126			−0.133		0.129	
		(−0.622)		(0.470)			(−0.671)		(0.483)	
*Creative*		0.002		0.001			0.002		0.001	
		(1.270)		(0.493)			(1.284)		(0.477)	
*Constant*		4.384 ***		4.837 ***			4.479 ***		4.903 ***	
		(5.921)		(4.701)			(6.033)		(4.761)	
*Year fixed effect*		Yes		Yes			Yes		Yes	
*Industry fixed effect*		Yes		Yes			Yes		Yes	
*Province fixed effect*		No		Yes			No		Yes	
*Observations*		4137		4137			4137		4137	
*Adj.R^2^*		0.310		0.328			0.310		0.329	

Note: Parentheses are t-statistics based on standard errors. ***, **, and * denote statistical significance at the 1%, 5%, and 10% levels, respectively.

**Table 9:** DID regression results for the impact of COVID-19 on analyst site visits and CEID in Wuhan and other cities in China.

Dep. EID	(1)	(2)	(3)	(4)
*Treatment*	−0.455 **	−0.238	−0.113	−0.243
	(−2.354)	(−1.497)	(−0.348)	(−0.579)
*Post*	4.073 ***	8.088 ***	8.033 ***	8.013 ***
	(21.844)	(33.420)	(33.218)	(32.623)
*Treatment×Post*	−1.618 ***	−2.360 ***	−2.187 ***	−1.738 ***
	(−8.657)	(−10.797)	(−10.986)	(−5.909)
*Instshr*		2.565 ***	2.912 ***	2.967 ***
		(5.028)	(5.616)	(5.265)
*ROA*		4.203 **	2.440 *	1.615
		(2.672)	(1.779)	(1.173)
*Age*		0.211	0.200	0.193
		(1.611)	(1.456)	(1.335)
*Big4*		−0.455	−0.451	−0.169
		(−1.184)	(−1.100)	(−0.457)
*TobinQ*		−0.100 ***	−0.084 ***	−0.087 ***
		(−7.844)	(−6.590)	(−6.676)
*Growth*		0.016	−0.008	−0.023
		(0.100)	(−0.056)	(−0.164)
*Meet_nmb*		0.194 ***	0.189 ***	0.195 ***
		(9.630)	(9.983)	(9.629)
*Leverage*		−0.346	−0.282	−0.410
		(−1.095)	(−0.816)	(−1.166)
*MSP*		1.573 ***	1.549 ***	1.529 ***
		(3.356)	(3.070)	(3.178)
*Market*		0.122	0.083	−0.122
		(0.956)	(0.682)	(−1.679)
*GDP_dum*		−0.651	−0.418	0.376
		(−1.478)	(−1.089)	(0.575)
*Creative*		0.002	0.007	0.026 *
		(0.116)	(0.526)	(1.994)
*Constant*	3.909 ***	−2.764 ***	−1.616	−2.251 **
	(28.193)	(−3.983)	(−1.526)	(−2.719)
*Year fixed effect*	No	Yes	Yes	Yes
*Industry fixed effect*	No	No	Yes	Yes
*Province fixed effect*	No	No	No	Yes
*Observations*	3584	3584	3584	3584
*Adj.R^2^*	0.107	0.467	0.490	0.497

Note: Parentheses are t-statistics based on standard errors. ***, **, and * denote statistical significance at the 1%, 5%, and 10% levels, respectively.

## Data Availability

The data used to support the findings of this study are available from the corresponding author upon request.

## Appendix A. Definitions of Variables

**Variables**	**Definition**
Explanatory variable	
*EID*	*EID* is scored according to 10 items about environmental information from a listed corporation’s annual report. Monetary information is awarded 3 points, specific non-monetary information is awarded 2 points, general non-monetary information is awarded 1 point, and no information is awarded 0 points (see Equation (1) in detail).
*EID_Soft*	Soft information includes three categories: vision and strategy, environmental measures, and public welfare activities related to the environment. The method for calculation is the same as for *EID*.
*EID_Hard*	Hard information includes environmental management systems, the reliability and credibility of environmental information, expenditure on environmental technology and investments, resource consumption and pollution control, important environmental problems and types of influence, and improvement in environmental performance. The method for calculation is the same as for *EID*.
Independent variable	
*Visit_dum*	Dummy variable that equals 1 if the corporation receives at least one site visit in the current year and is otherwise 0.
*Visit_nmb*	The natural logarithm of the total number of analyst site visits received by the corporation of the year plus one.
Firm-level variables	
*Instshr*	The shareholding ratio of institutional investors.
*ROA*	Income before extraordinary items is scaled by average total assets at the end of the period.
*Age*	The number of years since IPO/10.
*Big4*	Dummy variable that equals 1 if the firm is audited by a Big 4 accounting firm and is otherwise 0.
*TobinQ*	The ratio of the market value of a corporation’s equity and liabilities to its corresponding book values.
*Growth*	Sales growth equals the increase in the rate of the main business revenue. (Current operating income—Previous period’s operating income)/Previous period’s operating income.
*Meet_nmb*	Number of board meetings.
*Leverage*	The ratio of liabilities to assets.
*MSP*	The proportion of the total number of shares held by the board of directors, the board of supervisors, and senior executives in the total number of shares of the corporation.
*Duality*	Dummy variable that equals 1 if the chairman and general manager holding a concurrent post and otherwise it is 0.
*Shrcr10*	Sum of the top 10 major shareholders’ holding ratios of the corporation.
*Agent*	Agency cost is calculated as management expenses divided by operating income.
*Media*	The natural logarithm of the total number of news reports in the “Financial News Database of Chinese Listed Companies” of CNRDS plus one.
State-level variables	
*GDP_dum*	Regional per capita gross domestic product compiled by the CSMAR database. Dummy variable that equals 1 if the GDP is more than the median and is otherwise 0.
*Market*	Market level score for the place where the sample is located as determined by the Chinese Marketization Report [86].
*Creative*	The comprehensive utility value of regional innovation capability is obtained from the annual report of Regional Innovation Capacity of China.
*AQI*	Dummy variable that equals 1 if the analyst from a high air quality area visits a corporation from a low air quality area and is otherwise 0.
*Distance*	The geographical linear distance between the location of the visited corporations and the analyst’s institution. The geographical linear distance is calculated by latitude and longitude. Unit: 1000 km.
*Year*	Year dummy variable.
*Industry*	Industry dummy variable.
*Province*	Province dummy variable.

## References

[1] Economy E.C. (2007). The Great Leap Backward—The Costs of China’s Environmental Crisis. Foreign Aff..

[2] Zeng S., Liu H., Tam C., Shao Y. (2008). Cluster analysis for studying industrial sustainability: An empirical study in Shanghai. J. Clean. Prod..

[3] Du X. (2013). Does Religion Matter to Owner-Manager Agency Costs? Evidence from China. J. Bus. Ethics.

[4] Zhang Z., Zhang G., Song S., Su B. (2020). Spatial Heterogeneity Influences of Environmental Control and Informal Regulation on Air Pollutant Emissions in China. Int. J. Environ. Res. Public Health.

[5] Criado-Jiménez I., Fernández-Chulián M., Larrinaga-González C., Husillos-Carqués F.J. (2008). Compliance with Mandatory Environmental Reporting in Financial Statements: The Case of Spain (2001–2003). J. Bus. Ethics.

[6] Evans M.F., Gilpatric S.M., Liu L. (2009). Regulation with direct benefits of information disclosure and imperfect monitoring. J. Environ. Econ. Manag..

[7] Wang H., Bi J., Wheeler D., Wang J., Cao D., Lu G., Wang Y. (2004). Environmental performance rating and disclosure: China’s GreenWatch program. J. Environ. Manag..

[8] Zeng S.X., Xu X.D., Yin H.T., Tam C.M. (2012). Factors that Drive Chinese Listed Companies in Voluntary Disclosure of Environmental Information. J. Bus. Ethics.

[9] Zhu Q., Cordeiro J., Sarkis J. (2013). Institutional pressures, dynamic capabilities and environmental management systems: Investigating the ISO 9000—Environmental management system implementation linkage. J. Environ. Manag..

[10] Bowen F. (2014). After Greenwashing: Symbolic Corporate Environmentalism and Society.

[11] Yao S., Li S. (2018). Distance and government resource allocation: From the perspective of environmental information disclosure policy change. Appl. Econ..

[12] Ji Z., Yu X., Yang J. (2020). Environmental information disclosure in capital raising. Aust. Econ. Pap..

[13] Zheng Y., Ge C., Li X., Duan X., Yu T. (2020). Configurational analysis of environmental information disclosure: Evidence from China’s key pollutant-discharge listed companies. J. Environ. Manag..

[14] Liu X., Anbumozhi V. (2009). Determinant factors of corporate environmental information disclosure: An empirical study of Chinese listed companies. J. Clean. Prod..

[15] Wang H., Cao D., Wang J., Lu G. (2002). Environmental Information Disclosure: Theory and Practice.

[16] Hutton A.P., Lee L.F., Shu S.Z. (2012). Do Managers Always Know Better? The Relative Accuracy of Management and Analyst Forecasts. J. Account. Res..

[17] Cheng Q., Du F., Wang X., Wang Y. (2016). Seeing is believing: Analysts’ corporate site visits. Rev. Account. Stud..

[18] Han B., Kong D., Liu S. (2018). Do Analysts Gain an Informational Advantage by Visiting Listed Companies?. Contemp. Account. Res..

[19] Chen X., Cheng C.S.A., Xie J., Yang H. (2022). Private communication and management forecasts: Evidence from corporate site visits. Corp. Gov. Int. Rev..

[20] Yu F. (2008). Analyst coverage and earnings management. J. Financ. Econ..

[21] Huang C.-L., Kung F.-H. (2010). Drivers of Environmental Disclosure and Stakeholder Expectation: Evidence from Taiwan. J. Bus. Ethics.

[22] Brown L.D., Call A.C., Clement M.B., Sharp N.Y. (2015). Inside the “Black Box” of Sell-Side Financial Analysts. J. Account. Res..

[23] Wang K., Jiang W. (2019). Brand Equity and Firm Sustainable Performance: The Mediating Role of Analysts’ Recommendations. Sustainability.

[24] Bushee B.J., Jung M.J., Miller G.S., Ross S.M., Sandberg J. (2011). Conference Presentations and the Disclosure Milieu. J. Account. Res..

[25] Mayew W.J., Sharp N.Y., Venkatachalam M. (2013). Using earnings conference calls to identify analysts with superior private information. Rev. Account. Stud..

[26] Green T.C., Jame R., Markov S., Subasi M. (2014). Access to management and the informativeness of analyst research. J. Financ. Econ..

[27] Solomon D., Soltes E. (2015). What Are We Meeting For? The Consequences of Private Meetings with Investors. J. Law Econ..

[28] Bushee B.J., Jung M.J., Miller G.S. (2017). Do Investors Benefit from Selective Access to Management?. J. Financ. Rep..

[29] Chung K.H., Jo H. (1996). The Impact of Security Analysts’ Monitoring and Marketing Functions on the Market Value of Firms. J. Financ. Quant. Anal..

[30] Bae K.-H., Stulz R.M., Tan H. (2008). Do local analysts know more? A cross-country study of the performance of local analysts and foreign analysts. J. Financ. Econ..

[31] Jiang X., Yuan Q. (2018). Institutional investors’ corporate site visits and corporate innovation. J. Corp. Financ..

[32] Bowen R.M., Dutta S., Tang S., Zhu P.C. (2018). Inside the “Black Box” of Private In-House Meetings. Rev. Account. Stud..

[33] Yang Y., Shen L., Li Y., Li Y. (2022). The Impact of Environmental Information Disclosure on Environmental Governance Satisfaction. Sustainability.

[34] Darrell W., Schwartz B.N. (1997). Environmental disclosures and public policy pressure. J. Account. Public Policy.

[35] Cho C.H., Patten D.M. (2007). The role of environmental disclosures as tools of legitimacy: A research note. Account. Organ. Soc..

[36] Patten D.M. (2002). Media exposure, public policy pressure, and environmental disclosure: An examination of the impact of tri data availability. Account. Forum.

[37] Clarkson P.M., Li Y., Richardson G.D., Vasvari F.P. (2008). Revisiting the relation between environmental performance and environmental disclosure: An empirical analysis. Account. Organ. Soc..

[38] Ahmad N.N.N., Mohamad N.A. (2014). Environmental Disclosures by the Malaysian Construction Sector: Exploring Extent and Quality. Corp. Soc. Responsib. Environ. Manag..

[39] Meng X., Zeng S., Shi J.J., Qi G., Zhang Z. (2014). The relationship between corporate environmental performance and environmental disclosure: An empirical study in China. J. Environ. Manag..

[40] Fan L., Yang K., Liu L. (2020). New media environment, environmental information disclosure and firm valuation: Evidence from high-polluting enterprises in China. J. Clean. Prod..

[41] Yao S., Liang H. (2017). Firm location, political geography and environmental information disclosure. Appl. Econ..

[42] Iatridis G.E. (2013). Environmental disclosure quality: Evidence on environmental performance, corporate governance and value relevance. Emerg. Mark. Rev..

[43] Hackston D., Milne M.J. (1996). Some determinants of social and environmental disclosures in New Zealand companies. Account. Audit. Account. J..

[44] Yao S. (2020). Price pressure effects of short selling on environmental disclosure. Asia-Pac. J. Account. Econ..

[45] Brammer S., Pavelin S. (2006). Voluntary Environmental Disclosures by Large UK Companies. J. Bus. Financ. Account..

[46] de Villiers C., Naiker V., Van Staden C.J. (2011). The Effect of Board Characteristics on Firm Environmental Performance. J. Manag..

[47] Du X., Jian W., Zeng Q., Du Y. (2014). Corporate Environmental Responsibility in Polluting Industries: Does Religion Matter?. J. Bus. Ethics.

[48] Gupta S., Goldar B. (2005). Do stock markets penalize environment-unfriendly behaviour? Evidence from India. Ecol. Econ..

[49] Du X. (2015). How the Market Values Greenwashing? Evidence from China. J. Bus. Ethics.

[50] Dasgupta S., Laplante B., Wang H., Wheeler D. (2002). Confronting the Environmental Kuznets Curve. J. Econ. Perspect..

[51] O’Brien P.C., Bhushan R. (1990). Analyst Following and Institutional Ownership. J. Account. Res..

[52] Chen J., Ding R., Hou W., Johan S. (2016). Do Financial Analysts Perform a Monitoring Role in China? Evidence from Modified Audit Opinions. Abacus.

[53] Dyck A., Volchkova N., Zingales L. (2008). The Corporate Governance Role of the Media: Evidence from Russia. J. Financ..

[54] Patten D.M. (2002). The relation between environmental performance and environmental disclosure: A research note. Account. Organ. Soc..

[55] Cormier D., Magnan M. (2003). Environmental reporting management: A continental European perspective. J. Account. Public Policy.

[56] Xu Y.Y., Hong J.Q., Cao X.W. (2015). The Characteristic of Listed Companies of China and Securities Analysts’ Field Studies. Rev. Invest. Stud..

[57] Qi F., Li T. (2018). Study of Environmental Information Disclosure under the Hypothesis of Political Cost-Based on PM2.5 Burst Event. J. Financ. Econ..

[58] Dong R., Fisman R., Wang Y., Xu N. (2021). Air pollution, affect, and forecasting bias: Evidence from Chinese financial analysts. J. Financ. Econ..

[59] Zhang Z., Zhang G., Su B. (2022). The spatial impacts of air pollution and socio-economic status on public health: Empirical evidence from China. Socio-Econ. Plan. Sci..

[60] Jensen M.C., Meckling W.H. (1976). Theory of the Firm: Managerial Behavior, Agency Costs, and Ownership Structure. J. Financ. Econ..

[61] Wiseman J. (1982). An evaluation of environmental disclosures made in corporate annual reports. Account. Organ. Soc..

[62] Al-Tuwaijri S.A., Christensen T.E., Hughes K.E. (2004). The relations among environmental disclosure, environmental performance, and economic performance: A simultaneous equations approach. Account. Organ. Soc..

[63] Cormier D., Magnan M., Van Velthoven B. (2005). Environmental disclosure quality in large German companies: Economic incentives, public pressures or institutional conditions?. Eur. Account. Rev..

[64] Zeng S., Xu X., Dong Z., Tam V.W. (2010). Towards corporate environmental information disclosure: An empirical study in China. J. Clean. Prod..

[65] Patten D.M. (1992). Intra-industry environmental disclosures in response to the Alaskan oil spill: A note on legitimacy theory. Account. Organ. Soc..

[66] Solomon J.F., Solomon A. (2006). Private social, ethical and environmental disclosure. Account. Audit. Account. J..

[67] Xu X.D., Zeng S.X., Tam C.M. (2012). Stock Market’s Reaction to Disclosure of Environmental Violations: Evidence from China. J. Bus. Ethics.

[68] Li B., Gao F., Zeng Y.T. (2020). Impact of Air Pollution on Corporate Environmental Information Disclosure: Evidence from China. J. Environ. Prot. Ecol..

[69] Zhang Z., Zhang G., Li L. (2022). The spatial impact of atmospheric environmental policy on public health based on the mediation effect of air pollution in China. Environ. Sci. Pollut. Res..

[70] Zheng S., Cao C.-X., Singh R.P. (2014). Comparison of ground based indices (API and AQI) with satellite based aerosol products. Sci. Total Environ..

[71] Joe J.R., Louis H., Robinson D. (2009). Managers’ and Investors’ Responses to Media Exposure of Board Ineffectiveness. J. Financ. Quant. Anal..

[72] Bushee B.J., Core J.E., Guay W., Hamm S.J. (2010). The Role of the Business Press as an Information Intermediary. J. Account. Res..

[73] Barber B., Lehavy R., McNichols M., Trueman B. (2001). Can Investors Profit from the Prophets? Security Analyst Recommendations and Stock Returns. J. Financ..

[74] Xue J., He Y., Liu M., Tang Y., Xu H. (2021). Incentives for Corporate Environmental Information Disclosure in China: Public Media Pressure, Local Government Supervision and Interactive Effects. Sustainability.

[75] Brammer S., Pavelin S. (2004). Voluntary social disclosures by large UK companies. Bus. Ethics.

[76] Porter M.E., Kramer M.R. (2006). The Link Between Competitive Advantage and Corporate Social Responsibility. Harvard. Bus. Rev..

[77] Aerts W., Cormier D. (2009). Media legitimacy and corporate environmental communication. Account. Organ. Soc..

[78] Patten D.M., Trompeter G. (2003). Corporate responses to political costs: An examination of the relation between environmental disclosure and earnings management. J. Account. Public Policy.

[79] Wen Z., Ye B. (2014). Analyses of Mediating Effects: The Development of Methods and Models. Adv. Psychol. Sci..

[80] Fang L., Peress J. (2009). Media Coverage and the Cross-section of Stock Returns. J. Financ..

[81] Chahine S., Mansi S., Mazboudi M. (2015). Media news and earnings management prior to equity offerings. J. Corp. Financ..

[82] Tsileponis N., Stathopoulos K., Walker M. (2020). Do corporate press releases drive media coverage?. Br. Account. Rev..

[83] Shi C., Xu T., Ying Z., Li H. (2022). How Policy Mix Choices Affect the COVID-19 Pandemic Response Outcomes in Chinese Cities: An Empirical Analysis. Int. J. Environ. Res. Public Health.

[84] Fang H., Wang L., Yang Y. (2020). Human Mobility Restrictions and the Spread of the Novel Coronavirus (2019-nCoV) in China. J. Public Econ..

[85] Dong M., Zhou C., Zhang Z. (2022). Analyzing the Characteristics of Policies and Political Institutions for the Prevention and Control Governance of the COVID-19 Pandemic: Evidence from China. Int. J. Environ. Res. Public Health.

[86] Fan G., Wang X.L., Zhu H.P. (2014). China’s Marketization Index: A Report on the Relative Progress of Marketization in Different Regions.

